# Calcium Imaging in Drug Discovery for Psychiatric Disorders

**DOI:** 10.3389/fpsyt.2020.00713

**Published:** 2020-07-23

**Authors:** Saurav Seshadri, Daniel J. Hoeppner, Katsunori Tajinda

**Affiliations:** Neuroscience, La Jolla Laboratory, Astellas Research Institute of America LLC, San Diego, CA, United States

**Keywords:** autism, schizophrenia, sociability, cognition, drug discovery, calcium imaging, GCaMP, endoscopy

## Abstract

The past 5 years have seen a sharp increase in the number of studies using calcium imaging in behaving rodents. These studies have helped identify important roles for individual cells, brain regions, and circuits in some of the core behavioral phenotypes of psychiatric disorders, such as schizophrenia and autism, and have characterized network dysfunction in well-established models of these disorders. Since rescuing clinically relevant behavioral deficits in disease model mice remains a foundation of preclinical CNS research, these studies have the potential to inform new therapeutic approaches targeting specific cell types or projections, or perhaps most importantly, the network-level context in which neurons function. In this mini-review, we will provide a brief overview of recent insights into psychiatric disease-associated mouse models and behavior paradigms, focusing on those achieved by cellular resolution imaging of calcium dynamics in neural populations. We will then discuss how these experiments can support efforts within the pharmaceutical industry, such as target identification, assay development, and candidate screening and validation. Calcium imaging is uniquely capable of bridging the gap between two of the key resources that currently enable CNS drug discovery: genomic and transcriptomic data from human patients, and translatable, population-resolution measures of brain activity (such as fMRI and EEG). Applying this knowledge could yield real value to patients in the near future.

## Introduction

Drug discovery for brain disorders is a uniquely challenging endeavor (1, 2). One reason for this is that the brain’s output, i.e. behavioral responses to sensory input, is not sufficiently explained by genetics (3), as it is the result of complex computations performed by a vast network of neurons distributed in functionally distinct brain regions. To make progress towards new treatments for psychiatric disorders, it is crucial to understand brain functioning at the level of these neural circuits (4).

Action potentials (APs), electrical signals relayed between neurons *via* local and long-distance synaptic connections, are the mechanism by which neurons integrate and transmit information. While individual neurons’ firing patterns can be highly variable, at the population level, AP firing is reliable enough to form the basis for information coding in the brain (5). These population codes can be spatial, temporal, or both, with information represented by the subset or ensemble of active neurons, fluctuations in firing rates, or correlated activity between neurons (6). While many methods exist to record this activity, several lines of evidence suggest that cellular resolution is necessary to truly understand circuit functioning. While neurons exhibit diverse stimulus selectivity, behavioral output can often be reliably predicted by the activity of groups of individual cells (7). Inducing AP firing in specific neurons can disrupt a range of cognitive processes, including spatial navigation (8) and social behavior (9). Conversely, stimulating APs in specific subsets of neurons can be sufficient to elicit complex, clinically relevant behavioral responses, such as improved decision making (10) and mood (11).

Given their importance to brain functioning, it is unsurprising that many brain disorders can be attributed to dysfunctional AP firing. For example, studies using mouse models (12) or human induced pluripotent stem cells (13) have revealed that ion channel mutations associated with epilepsy also alter AP properties. While these changes are profound and detectable in individual cells, the pathophysiology underlying psychiatric disorders is less straightforward. This complexity begins at the genetic level: although diseases like schizophrenia and autism are highly heritable, much of this heritability is driven by common variants with small effect sizes (14, 15), whose individual impact on neuronal activity may be difficult to predict or detect. Copy number variants and *de novo* mutations, which can be more penetrant and form the basis for several disease models, are relatively rare but are enriched in patients with psychiatric disorders (16–18).

How do these varied risk factors ultimately produce a relatively consistent clinical phenotype? One explanation is that they converge on common pathways, and several lines of evidence suggest that specific neural circuit components represent this point of convergence (19). Functional genomics studies indicate that the genetic burden in psychiatric disorders disproportionately affects distinct excitatory neuron subpopulations (20–22). Other cell types, such as inhibitory fast-spiking interneurons, are implicated by postmortem and physiological reports (23, 24). In addition, gene ontology studies consistently point to impaired synapse formation as a core feature of schizophrenia and autism (25–27). Observations of altered excitatory-inhibitory balance (28, 29) and brain connectivity (30, 31) in psychiatric patients substantiate these inferences. If convergence underlies pathogenesis, we would expect to see similar circuit-level deficits in models with different etiologies. In fact, while the above studies are based on human data, similar commonly shared deficits are also observed at the level of neuroanatomy (32), population physiology (33), and cellular resolution physiology (34, 35) in mouse models of disease. Overall, these findings suggest that studying pathophysiology in neural circuits, ideally with cellular resolution and/or cell type specificity, is likely to provide mechanistic insight into psychiatric disorders.

**Figure 1:**
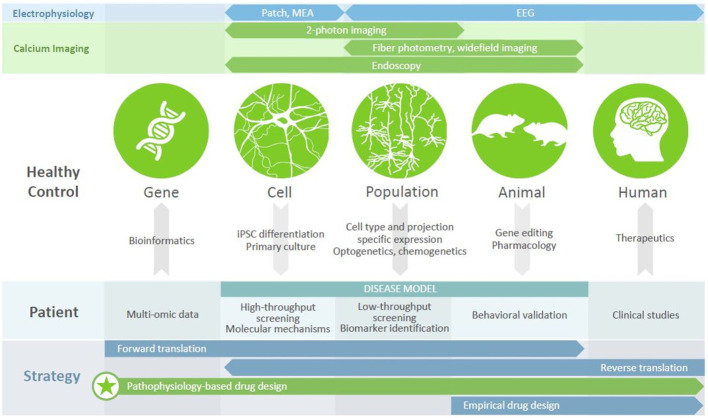
Calcium imaging in disease modeling and drug discovery. Different modalities (top panels) provide access to different units of analysis (circles), with calcium imaging bridging the cell, population, and animal levels. Numerous tools exist (arrows) to generate disease models of varying complexity, each of which has utility in drug discovery efforts (middle panel) that can be revealed using calcium imaging. Leveraging these platforms, to understand the mechanisms by which disease-related genes bring about clinical symptoms, can underpin a pathophysiology-based approach to drug design (bottom panel), which may be the key to discovering new therapeutics for patients with psychiatric disorders.

In recent years, imaging intracellular calcium dynamics using genetically encoded calcium indicators (GECIs) has emerged as the method of choice to study activity in neural circuits. Current-generation GECIs can track the firing of single APs in individual cells, in awake, behaving mice (36). GECIs enable a range of experimental designs (**Figure 1**). Firstly, recording and subsequent analysis can be restricted to specific neuronal populations, defined by marker enrichment using Cre-dependent expression, functionality using inducible expression, or projection targets using retrograde virus transport. Secondly, activity can be recorded with population resolution (using fiber photometry or widefield imaging), supporting comparisons to LFP (local field potential) or EEG (electroencephalography) recordings, or with cellular resolution (using 1- or 2-photon fluorescent microscopy). While methods have been developed to allow head-fixed mice to perform several complex behavioral paradigms (37, 38), the advent of head-mounted cameras (39, 40) has allowed for recording brain activity during more naturalistic, disease-relevant behaviors such as reciprocal social interactions; when combined with endoscopy, this approach also allows the visualization of deep brain structures (41). Calcium imaging therefore represents a versatile and powerful approach to understanding neural circuit functioning.

Disease-associated genes, as well as candidate drugs, often impact intracellular signaling, membrane properties, cytoarchitecture, or other features of individual cells. By gathering cellular resolution data at population scale, calcium imaging allows us observe the effect of genetic modulation on a computationally relevant readout (i.e., neuronal activity), as well as to contextualize this activity in the larger network, behavioral state, or translatable signal. In the following sections, we will discuss recent insights from this approach into psychiatric disease models and behavior paradigms, and how these efforts can support drug discovery.

## Calcium Imaging During Spatial Cognition

Place cell dynamics in CA1 pyramidal neurons are well-established and readily detectable (42), leading them to be one of the first physiological phenomena to be studied *in vivo* using calcium imaging (43). These cells support navigation by reporting spatial location, and may be involved in cognitive processes such as episodic memory, which is disrupted in psychiatric disorders (44, 45). They represent a potential point of convergence for schizophrenia-associated genes (21), and have been implicated in mouse models of autism (46). In humans, altered hippocampal volume and functional connectivity is consistently observed in psychiatric disorders (47), but individual cells’ activity patterns cannot be detected using EEG. Three key factors therefore combine to make CA1 pyramidal cells a prime candidate for study using calcium imaging: existence of an expected activity pattern (providing a reliable basis for comparison between wild type and transgenic mice), disease relevance, and a gap in understanding of cellular pathophysiology.

Investigating place cell functioning in mouse models of schizophrenia and autism has yielded interesting results. Patients with microdeletions in chromosome 22q11.2 are at high risk for schizophrenia, and show cognitive impairment that is recapitulated in a mouse model (48). These mice show altered place field dynamics, including reduced stability, and fewer, narrower fields (49). Some of these phenotypic alterations, such as a reduction in place fields per cell, were also seen in DISC-1 mutant mice, another genetic schizophrenia model. In contrast, DISC-1 mutants also exhibited unique phenotypes, such as broader place fields (50). Fragile X syndrome (FXS), the most common monogenic form of autism, is caused by a loss of expression of the translational regulator FMRP, and FMR1 knockout mice provide a model for this disorder with good construct validity (51). In these mice, individual cells’ place tuning is intact, but coordinated activity of ensembles is significantly reduced (52). Interestingly, this instability correlates with impaired cognitive performance on a spatial learning task.

These findings illustrate how using calcium imaging in a forward translation approach, i.e. starting with a genetic perturbation and observing downstream effects, can provide mechanistic insight into disease. Place cell dynamics provide a robust platform to study how region 22q11.2 or FMRP target genes affect neuronal activity patterns associated with cognition. Using this platform to dissect the molecular mechanisms mediating the circuit level effects of these mutations could in turn identify routes to therapeutic intervention. More broadly speaking, understanding how different features of place cell dynamics distinguish schizophrenia models of different etiology, and how they correlate with cognitive performance, could support patient stratification and biomarker development respectively.

## Calcium Imaging During Social Behavior

Reverse translation, i.e. starting with a face-valid paradigm for a disease-relevant phenotype and attempting to understand the neural circuitry underlying it, can also be a fruitful strategy in translational research (53). Several factors make social behavior a good candidate for this strategy. Deficits in sociability and social cognition are a key diagnostic criterion and clinical endpoint for psychiatric disorders (54). Furthermore, while many aspects of these diseases (such as psychosis or intellectual disability) are difficult to model in mice, several paradigms exist to test social behaviors (55). Targeted perturbations have shown that these behaviors require the coordinated activity of several interconnected cortical and subcortical brain regions (56), but the neurons involved, their normal activity patterns, and disease-state abnormalities are not known. Recent studies have used calcium imaging in inventive ways to address this question.

Several studies have used a fiber photometry approach to study region and cell type-specific activity patterns during social behavior. Although this is a population-resolution recording technique, these studies are able to provide cell-level insights by using cell type-specific or projection-specific expression to interrogate defined subpopulations of neurons. Labeling a genetically defined subpopulation of pyramidal neurons in the medial prefrontal cortex (mPFC) revealed that dopamine D2 receptor positive, subcortically projecting neurons are recruited during social exploration, and that this recruitment is attenuated in a mouse model of autism (9). A retrograde viral targeting approach was used to show that dopaminergic projections from the ventral tegmental area to D1 receptor expressing neurons in the nucleus accumbens (NAc), rather than the mPFC, support social behavior (57). Dopaminergic projections from the dorsal raphe nucleus also bidirectionally control sociability in an emotional state dependent manner (58). Multiple studies have used parvalbumin-Cre mice to obtain fast-spiking interneuron specific expression, and have consistently found that increased activity in these cells is associated with social behavior (59–61) [but not emotional state discrimination, which is driven by somatostatin expressing interneurons (62)]. Building on this insight, specific stimulation of fast-spiking neurons is sufficient to recover social behavior deficits of diverse etiology, including autism-like cortical hyperexcitability (63), fear conditioning (64), genetic alteration (61, 65), and juvenile social isolation (59).

Calcium imaging using endoscopy has corroborated many of these findings, while also revealing more subtle activity patterns during social behavior. Cellular resolution imaging of mPFC neurons confirmed that specific subpopulations drive social behavior, and that the NAc is an important target for this information (66). Imaging in the amygdala and hypothalamus confirms responses to social stimuli in these regions, and demonstrate that this activity encodes important social features, such as the gender of the target mouse (67, 68). In most cases, cellular resolution allows for a more nuanced understanding information coding—for example, mPFC neurons encode a combination of social and spatial information, and exhibit diverse responses (e.g. increased or decreased activity) to social stimuli (66, 69). Neuronal responses are also often context-dependent, with varying levels of activity during different behavioral phases (e.g. approach *vs.* withdrawal) (57).

In addition to refining our basic understanding of neural circuitry and behavior, these findings have novel and practical implications for drug development. Complex, de-correlated activity patterns may not be visible in population resolution signals, and suggest that a blanket enhancement or inhibition of activity (as may be the outcome of many pharmacological interventions) may not improve social cognition. Drugs with use-dependent mechanisms, such as allosteric modulators, or drugs that target molecules that are preferentially expressed in relevant neuronal populations, such as certain neurotransmitter receptors, may therefore be more effective. Similarly, projection-specific deficits may not be visible in postmortem tissue, and identifying these pathways could point to potential targets for emerging treatment modalities, such as transcranial magnetic stimulation or gene therapy.

## Calcium Imaging During Sensory Processing

Even with carefully chosen transgenic models and behavioral paradigms, translatability remains a major challenge to drug discovery efforts. EEG can overcome this challenge by recording the same signals in mice and humans. Several brain activity endophenotypes have been identified in patients with psychiatric disorders using this approach (70), and these are often replicated in corresponding mouse models (71). Despite being a population resolution measure, some EEG endophenotypes have been linked to underlying deficits in specific neuronal subpopulations, such as gamma-band oscillatory power and fast-spiking interneurons (24, 72). Another class of deficits is in sensory processing, as measured using event-related potentials (ERPs). These sensory-evoked population signals are clearly altered in patients with psychiatric disorders, but a lack of understanding of the cellular underpinnings of these phenomena has made it difficult to use them to infer causal mechanisms.

A few recent studies have attempted to bridge this gap, by establishing disease-relevant sensory processing tests in mice and combining them with calcium imaging. The first study replicated a visual mismatch negativity test, often used as a biomarker in patients with schizophrenia, in mice (73). By combining this paradigm with LFP recordings, the authors confirmed that population-level signals evoked by deviant and redundant stimuli correspond with neuronal activity in layer 2/3 of the cortex, providing ground truth evidence for a crucial assumption of EEG recordings in humans. They went on to show that non-fast spiking interneurons modulate this effect at the local microcircuit level. This finding was supported by experiments in schizophrenia model mice, including the 22q11.2 microdeletion model, which showed disorganized population activity that was not reproduced by chemogenetic inhibition of fast-spiking interneurons (35). A separate study focused on activity in V1 during a visual discrimination task, in which Fmr1-KO mice were impaired (74). Importantly, the authors confirmed that patients with FXS showed deficits in the same test. Using calcium imaging to separately observe neuronal subpopulations, they found deficits in orientation tuning in pyramidal cells and in evoked activity in fast-spiking interneurons; the visual discrimination impairment was reversed by cell type specific chemogenetic stimulation of fast-spiking interneurons.

The above studies show that with careful validation in patients and recording of multimodal physiological signals, it is possible to establish informative tests of sensory processing that can provide convincing insight into human brain circuit functioning. By taking advantage of the increased accessibility of neural circuitry in mice, and the cellular resolution afforded by calcium imaging, these experiments have pinpointed circuit components and activity patterns that could be promising targets for treatment. Expanding this approach to other biomarkers, particularly those that may stratify patients (75), should be a priority for translational research.

Finally, we should note that cognitive impairment, which has been linked to sensory processing deficits (76, 77), is a critical component of many psychiatric diseases (54). Several high-quality studies have used calcium imaging to observe population activity during cognitive tasks (78, 79); however, given the diversity and complexity of behavioral tests for cognitive impairment, it can be challenging to extrapolate these findings to disease states. For example, the studies cited above used the T-maze and novel object test, but designed their tasks to test decision-making and object memory respectively [rather than working memory, a domain of psychiatric disorders (80) for which these tests are often used (81, 82)]. As the field progresses, we hope to see more examples of calcium imaging applied to cognitive behavior testing in psychiatric disease models.

## Potential Impact on Drug Discovery Research

Over the past decade, large-scale efforts have generated sufficiently powered cohorts to identify significantly disease-associated genes, which should, in theory, be the key to selecting new drug targets. However, there are three fundamental gaps that need to be cleared before we can understand how a “hit” gene may impact disease: first, how the gene’s functioning affects network activity, second, how this network activity supports a disease-related behavioral domain, and third, whether this network activity is in fact disrupted in the disease, and if rescuing it recovers the disease-related behavior. Answering these questions represents a “pathophysiology-based” approach to drug discovery (83). Current treatments for psychiatric disorders, where available, were discovered empirically and are poorly understood, show limited efficacy, and have numerous side effects. A better understanding of pathophysiological mechanisms would allow more rational, and eventually more successful, drug design (4).

With this goal in mind, there is a compelling case for making calcium imaging central to modern neuroscience drug discovery programs. It can dramatically increase the throughput of measuring neuronal activity *in vitro*, using primary cultures from transgenic mice or patient-derived differentiated iPSCs. This approach can answer the question of how disease-associated mutations impact neuronal activity, by using patient cells containing the mutation, or introducing the mutation or its corresponding gain or loss of function into rodent neurons (by gene editing or viral transduction). Alternatively, it can be used to screen compound effects on physiology, with the advantage of also allowing unambiguous matching of neuronal activity to gene expression or other cellular phenotypes by post-hoc immunostaining. These effects can then be validated *in vivo*, enabling better tissue preservation and higher cell yields than the standard alternative of slice electrophysiology, as well as providing the ability to do long-term drug treatments. With calcium imaging, it is thus possible to build a screening pipeline that integrates molecular biology and physiology, and spans *in vitro* and *in vivo* models using the same modality.

Patients with psychiatric diagnoses are not a homogenous population (75), and a critical challenge in developing new therapies is to understand the differences between subgroups of patients and identify likely responders. Biomarkers of brain activity are necessary in this effort, and calcium imaging has the potential to reveal more sensitive and informative measures of circuit functioning than those currently available. Due to the invasive nature of calcium imaging, these cellular resolution biomarkers cannot be recorded in humans using current technology; however, several strategies can be used to obtain translatable insights. Firstly, recordings using a translatable modality can be made in parallel, to directly link cell and population resolution phenotypes. For example, a study in a mouse model of epilepsy found changes in calcium activity that accompanied behavioral and EEG manifestation of seizures (84); in fact, altered calcium dynamics preceded EEG detection of seizure onset, and provided a potential mechanism linking seizure activity with cellular damage. Second, models can be chosen or designed with sufficient construct validity that we can assume pathophysiological similarity between rodents and humans. Along these lines, recent studies have characterized network abnormalities in models of cocaine or alcohol dependence (85, 86), for which the etiology in mice and humans is similar; equivalent models for psychiatric disorders also exist (such as the Fmr1 knockout mice described above). Third, nonhuman primates are much more comparable to humans than mice. Transgenic marmosets expressing GECIs are now available (87), and can be used to model sophisticated, disease-relevant behaviors (88).

The last, and perhaps most powerful, strategy is to use existing clinically effective drugs to test the predictive validity of newly identified cellular resolution biomarkers. For example, a recent study using a rodent model of Parkinson’s disease (PD) identified cell type-specific, disease-associated activity patterns in distinct, genetically defined subpopulations of medium spiny neurons in the striatum (89). The authors found that clinically used treatments for PD rescued these patterns, with the greatest clinical efficacy associated with the drug that rescued altered activity patterns to the greatest extent. This approach is profoundly beneficial, as it directly identifies the cell types and circuit abnormalities associated with disease, while simultaneously providing a platform to screen new compounds for improved clinical efficacy. Opportunities exist to apply a similar approach to psychiatric disorders—for example, dopaminergic signaling is known to be involved in the pathophysiology of schizophrenia, but different pathological deficits have been associated with different brain regions and symptoms (i.e. striatal hyperdopaminergia with psychosis and cortical hypodopaminergia with cognitive impairment) (90). The antipsychotic drug aripiprazole, at one point the best-selling drug in the country, is a partial agonist of D2R receptors, meaning that its hypothesized mechanism of action is to stimulate D2R activity in the cortex while simultaneously competitively inhibiting D2R activity in the striatum (91). This could be confirmed by using calcium imaging to directly record the activity of D2R-expressing neurons and their postsynaptic targets in these regions, following treatment with aripiprazole or other antipsychotic drugs. Identifying the activity patterns modulated by existing antipsychotics would provide valuable insight into the mechanisms underlying psychosis, some of which could be targets for more clinically effective, domain specific, or conceptually innovative treatments in the future. As with PD, this approach could also provide the tools needed (i.e. screening platforms based on sensitive, informative cellular resolution biomarkers) to enable pathophysiology-based discovery of new drugs.

## Conclusion

Calcium imaging is a powerful method to study patterns of activity in defined neural circuits during behavior, which may be critical to understanding brain dysfunction in psychiatric disorders. Exciting progress has been made in applying calcium imaging to psychiatric disease-relevant paradigms and models. The field has already yielded several insights linking gene functioning, cellular physiology, translatable biomarkers, and behavior; continuing to build on this progress could contribute to drug discovery in a meaningful way. Applied correctly, cellular resolution imaging can help generate and evaluate mechanistic hypotheses, screen drugs and targets, and ultimately provide a roadmap to more effective medications in the future.

## Author Contributions

SS drafted the manuscript. SS, DH, and KT contributed to manuscript revision, read and approved the submitted version.

## Funding

The authors are supported and funded by Astellas Research Institute of America, LLC.

## Conflict of Interest

All authors are employed by the company Astellas Research Institute of America LLC. The authors are supported and funded by Astellas Research Institute of America, LLC. The funder was not involved in the study design, collection, analysis, interpretation of data, or the writing of this article. The funder approved the decision to submit this article for publication.
